# Efficacy and safety of fenoldopam for the treatment of hypertensive crises in children with kidney disease: a retrospective study

**DOI:** 10.1007/s00467-024-06490-7

**Published:** 2024-09-09

**Authors:** Nicola Bertazza Partigiani, Serena Vigezzi, Davide Meneghesso, Matteo Tinnirello, Alessandra Rosalba Brazzale, Marco Daverio, Enrico Vidal

**Affiliations:** 1 Department for Women’s and Children’s Health, University of Padua, Padua, Italy; 2 Pediatric Nephrology Unit, Department for Women’s and Children’s Health, University-Hospital of Padua, Padua, Italy; 3 Department of Statistical Sciences, University of Padua, Padua, Italy; 4 Pediatric Intensive Care Unit, Department of Women’s and Children’s Health, University Hospital of Padua, Padua, Italy; 5 Department of Medicine (DMED), University of Udine, Udine, Italy

**Keywords:** Pediatric, Hypertension crisis, Fenoldopam, Chronic kidney disease, Acute kidney injury

## Abstract

**Background:**

Hypertensive crises in children represent critical medical situations characterized by severe hypertension and potential organ damage. Fenoldopam, a dopaminergic medication, offers a viable therapeutic option for managing such clinical scenarios. We aimed to evaluate efficacy and safety of fenoldopam in the management of hypertensive urgencies and emergencies.

**Methods:**

This retrospective analysis focused on pediatric patients affected by acute or chronic kidney disease, aged 1 month–18 years, admitted to the Pediatric Nephrology and the Pediatric Intensive Care Unit at University-Hospital of Padua, Italy, who presented with a hypertensive crisis treated with fenoldopam between March 2010 and December 2022.

**Results:**

The study included 74 patients with median age 10 years (interquartile range [IQR] 4–15 years) who received 102 fenoldopam infusions. Seventy-two percent were already receiving antihypertensive treatment before admission. In all cases, fenoldopam was associated with a reduction of blood pressure (BP) after 8 h of treatment, but in 87% of patients reduction of the initial mean arterial pressure (MAP) was higher than 25% of calculated drop pressure. MAP normalized in 26% of cases after 24 h and in 35% after 48 h. Occurrence of hypotension was 7%, while hypokalemia was observed in 13% of cases. Patients who presented a MAP reduction not exceeding 25% of calculated drop pressure received a lower median fenoldopam dose (0.2 mcg/kg/min; IQR 0.1–0.2) compared with patients having a MAP reduction > 25% of calculated drop pressure (0.4 mcg/kg/min; IQR 0.2–0.6; *p* = 0.002).

**Conclusions:**

Fenoldopam seems effective and safe for the treatment of hypertensive crises in children with kidney disease, at a starting dose of 0.2 mcg/kg/min. Strict BP monitoring is required to identify possible excessive drop pressure in the first hours of infusion.

**Graphical abstract:**

**Figure:**
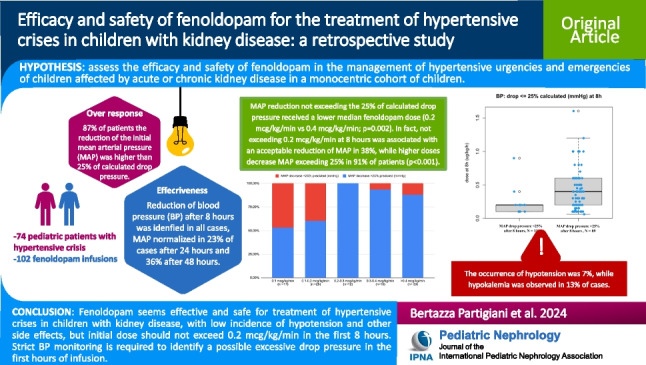
A higher resolution version of the Graphical abstract is available as Supplementary information

**Supplementary Information:**

The online version contains supplementary material available at 10.1007/s00467-024-06490-7.

## Background

According to the recommendations from the European Society of Hypertension (ESH), intravenous treatment should be used when addressing a hypertensive emergency [1–5]. Within this context, sodium nitroprusside (SNP) emerges as the foremost primary therapeutic choice. However, the prolonged use of SNP carries certain risks, notably the potential for cyanide toxicity especially in patients with kidney disease [6, 7]. Nicardipine, an intravenous dihydropyridine calcium ion influx inhibitor, is also largely used in children to manage hypertension, leveraging its rapid onset and titratable infusion for precise blood pressure (BP) control and it is one of the most emerging drugs for its safety and efficacy profiles as treatment for hypertensive crises [8].

 Twenty to 70% of children with acute kidney injury (AKI) or chronic kidney disease (CKD) present uncontrolled hypertension [9–12], thus requiring treatment with medications having a good kidney safety profile [9, 13–15].

Fenoldopam is an antihypertensive medication with vasodilatory properties, particularly targeting the kidney blood vessels, and it has been employed in pediatric patients at high risk of developing AKI, with the aim of preventing its progression [16]. Its use has also been described in the management of hypertensive crises in children, with no strong level of evidence [17]. The drug is a selective dopamine-1 (DA-1) receptor agonist which primarily acts on the kidney arteries and promotes vasodilation of the kidney blood vessels, leading to increased blood flow. Furthermore, DA-1 receptors are situated in the proximal convoluted tubules and cortical collecting ducts. Upon activation, these receptors produce kidney arterial vasodilation, urinary sodium excretion, and an increase in urinary flow, with a subsequent decrease in systemic BP. Fenoldopam is known for its onset of action within 5 to 15 min, which is advantageous in situations where rapid BP control is required [16, 18].

Fenoldopam efficacy has been studied mostly in the adult population, whereas in children, the existing knowledge regarding the efficacy and safety of the drug is based on case series and some observational studies [19–21]. In this study, we aimed to evaluate whether the use of fenoldopam for the treatment of hypertensive urgencies or emergencies in children with kidney diseases is safe and effective.

## Methods

### Patients and data collection

This retrospective study encompasses a cohort of children aged between 1 month to 18 years of age who were diagnosed with AKI or CKD and received intravenous fenoldopam treatment for at least 8 h. The patients were admitted to the Pediatric Nephrology ward or the Pediatric Intensive Care Unit at the University Hospital of Padua, Italy, from March 2010 to December 2022 and received fenoldopam infusion.

Patient data collected for analysis included age, gender, body weight, underlying medical conditions, eGFR (calculated following the Bedside Schwartz formula), evidence of AKI or CKD at the time of fenoldopam infusion (based on KDIGO criteria) [22], pre-existing hypertension (defined as the use of at least one antihypertensive medication before admission), chronic use of calcineurin inhibitors (CNI), and signs of fluid overload (characterized by a 5% or more increase in baseline body weight, clinical manifestations of generalized edema, or pulmonary edema).

### Definitions

Fenoldopam infusion was considered in case of a hypertensive crisis, defined as:

- Hypertensive urgency: systolic blood pressure (SBP) or diastolic blood pressure (DBP) > 95th percentile + 30 mmHg for patient age, height, and sex without evidence of target organ damage [4].
- Hypertensive emergency: SBP or DBP > 95th percentile + 30 mmHg for their age, height, and sex with evidence of target organ damage [4].

The 95th percentile of BP was defined according to the ESH guidelines [4]. SBP and DBP were recorded both with invasive and non-invasive methods, depending on the patient's status. SBP and DBP were collected at the start of fenoldopam infusion, after 8, 24, and 48 h, and at the end of infusion. Furthermore, the dose of fenoldopam expressed in mcg/kg/min was recorded at 8, 24, and 48 h after the beginning of infusion; the maximal dosage and the duration of fenoldopam infusion were also registered. Mean arterial pressure (MAP) was calculated with a standard formula: DBP + 1/3 [SBP – DBP]; the 95th MAP percentile was calculated based on SBP and DBP reference data [4]. To make meaningful comparisons across the pediatric age range, BP standard deviation scores (SDS) were calculated for each subject [23].

The following BP thresholds were defined to assess fenoldopam efficacy: I. reduction of BP (SBP, DBP, MAP) not exceeding the 25% of calculated drop BP after 8 h of infusion. The desired drop in BP was defined as the difference between the BP at the start of fenoldopam infusion and the 95th BP percentile for age, height, and sex; II. MAP equal to or less than 95th percentile for age, height, and sex after 24 and 48 h of infusion.

The definition of hypotension during the treatment was defined as a BP < 5th percentile for patient age, height, and sex with or without symptoms requiring stopping of the infusion. A BP drop higher than 25% of the calculated drop BP after 8 h of treatment was also evaluated to assess the presence of potentially associated clinical conditions.

### Outcome measures

Primary outcome was a reduction of blood pressure (SBP, DBP, MAP) not exceeding the 25% of calculated drop BP after 8 h of infusion, and BP normalization at 24 or 48 h. We also calculated the maximum per-kilo dose of fenoldopam able to safely reduce the BP. In outcome evaluation, MAP was used to account for both systolic and diastolic values, thus making a comprehensive evaluation of BP status also in patients with a significant differential BP or in those having a significant variation of only one of either SBP or DBP.

We decided to use MAP instead SBP and DBP values in order to perform a comprehensive analysis of both hypertensive values to determine the efficacy of fenoldopam (i.e. in patients with an important differential BP, or in case of a significant reduction of only one of either SBP or DBP).

Lastly, we aimed to identify side effects including hypotension and hypokalemia. Evidence of target organ damage during fenoldopam infusion was considered as a marker of therapeutic inefficacy.

### Statistical analysis

Frequency distributions and proportions were used to summarize categorical variables. Numerical variables were summarized through means (± standard deviation) and medians (interquartile range [IQR]) according to their distribution. Hypotheses on continuous measurements were tested using two-sided Student’s t-tests, ANOVA, and their nonparametric counterparts such as Wilcoxon–Mann–Whitney tests. Fisher’s exact tests and Pearson’s Chi-squared tests were used to compare categorical variables according to the numerosity of the sample. All tests were carried out at the 5% significance level. Correspondingly, two-sided confidence intervals were computed at the 95% confidence level.

Multiple linear regression and logistic regression were used to assess the significance of potential predictors on continuous and binary responses, respectively. To evaluate treatment effect on BP at 8, 24 and 48 h, fenoldopam dose was considered as a modifying variable and all other baseline variables (including age, sex, underlying disease, eGFR, evidence of AKI or CKD, pre-existing hypertension and number of antihypertensive drugs, presentation as hypertensive urgency or emergency, and fluid overload) as possible predictors of therapeutic response. Broken-line regression was used to compute threshold values.

## Results

### Population characteristics

Seventy-four patients who received 102 fenoldopam infusions were included in the study. The population characteristics are presented in Table 1. Patients’ median age was 10 years (IQR 4–15 years) and 60% were male. Thirty-eight patients presented a previous kidney transplantation, and 20 patients were affected by a primary kidney disease. Eighty percent of patients had a pre-existing diagnosis of CKD, while 20% presented with AKI. In patients with CKD, 11 had an eGFR > 90 ml/min/1.73 m^2^, 2 had an eGFR between 60–89 ml/min/1.73 m^2^, 3 had an eGFR between 30–59 ml/min/1.73 m^2^, 5 had an eGFR between 15–29 ml/min/1.73 m^2^, and 53 (72%) patients exhibited kidney failure.

**Table 1:** Population characteristics at the start of fenoldopam infusion

Population characteristics	
*N*° patients	74
Sex	
Male	44 (60%)
Female	30 (40%)
Evidence of kidney disease	
AKI	15 (20%)
CKD	59 (80%)
Underlying disease in CKD group	*n* = 59
Kidney transplantation	35 (59%)
Primary kidney disease	17 (28%)
Rheumatological disease	1 (2%)
Oncological disease	3 (5%)
Other	3 (5%)
Underlying disease in AKI group	*n* = 15
Post-surgical intervention (liver transplantation, heart transplantation, other surgery)	5 (33%)
Kidney transplantation	3 (20%)
Primary kidney disease	3 (20%)
Rheumatological disease	1 (7%)
Oncological disease	1 (7%)
Other	2 (13%)
Kidney function (eGFR)	
> 90 ml/min/1.73 m^2^	11 (15%)
60–89 ml/min/1.73 m^2^	2 (2%)
30–59 ml/min/1.73 m^2^	3 (4%)
15–29 ml/min/1.73 m^2^	5 (7%)
< 15 ml/min/1.73 m^2^	53 (72%)
Pre-existing hypertension	53 (72%)
Number of antihypertensive drugs in therapy before the start of infusion	
1	11 (15%)
2	4 (5%)
3 or more	38 (52%)
CNI therapy	41 (55%)

*eGFR* Estimated glomerular filtration rate; *AKI* Acute kidney injury; *CKD* Chronic kidney disease; *CNI* Calcineurin inhibitors

Among patients with acute kidney dysfunction, 47% had stage I, 13% stage II and 40% stage III AKI. The etiology of AKI and CKD diseases are reported separately in Table 1.

Most of the patients (72%) were already receiving antihypertensive medications before their hospital admission. Within this group, 15% were treated with a single medication, 5% with two medications, and in 52% of patients more than two different antihypertensive drugs were prescribed, which were continued during fenoldopam infusion, unless needed for fasting. Forty-one percent of patients were treated with CNI. Eighty-three percent of patients experienced a systolic hypertensive crisis, while 17% had an isolated diastolic hypertensive crisis and 36% of patients exhibited both SBP and DBP that exceeded the 95th percentile by 30 mmHg for their age, height, and sex.

### Fenoldopam infusion characteristics

Fenoldopam infusions were initiated for treating hypertensive urgencies in 86% of cases, and emergencies in 14% of cases (Table 2). Among hypertensive emergencies, neurological involvement was the most common presentation, affecting 65% of cases, while cardiac (7%), ocular (14%), and external bleeding (14%) were less frequent. Four out of the 9 patients with neurological involvement during a hypertensive emergency were also treated with anti-seizure medications. Following resolution of the epileptic crisis, all patients required continuation of the fenoldopam therapy due to persistent BP elevation, with one patient exceeding the 95th percentile for age, height, and sex, and the remaining three exceeding the 95th percentile by an additional 30 mmHg.

**Table 2:** Fenoldopam infusion characteristics and efficacy

Fenoldopam infusions	
*N*° of infusions	102
Duration (h)	115 (76–302)
Median maximal dose administered (mcg/kg/min)	0.6 (0.4–1.4)
Type of hypertension crises indication	
Hypertensive urgency	88 (86%)
Hypertensive emergency	14 (14%)
Target organ involvement in hypertensive emergencies	
Neurologic	9 (65%)
Ocular	2 (14%)
External bleeding	2 (14%)
Cardiac	1 (7%)
Median dose administered (mcg/kg/min)	
After 8 h	0.3 (0.2–0.5)
After 24 h	0.4 (0.2–0.8)
After 48 h	0.5 (0.3–1.0)
Reduction of blood pressure after 8 h	
Less than 25% of calculate mean arterial blood pressure reduction	13 (13%)
Less than 25% of calculate systolic blood pressure reduction	17 (17%)
Less than 25% of calculate diastolic blood pressure reduction	23 (23%)
Normalization (< 95th percentile) of blood pressure	
After 24 h (*N* = 93)	
Mean arterial pressure	24 (26%)
Systolic blood pressure	18 (19%)
Diastolic blood pressure	31 (33%)
After 48 h (*N* = 89)	
Mean arterial pressure	31 (35%)
Systolic blood pressure	21 (24%)
Diastolic blood pressure	31 (35%)
At the end of infusion	
Mean arterial pressure	79 (77%)
Systolic blood pressure	68 (66%)
Diastolic blood pressure	81 (79%)
Surgical treatment of hypertension	7 (7%)

Furthermore, at the onset of the infusion, 43% of cases presented with fluid overload which was treated in all cases with loop diuretics or hemodialysis in patients refractory to diuretics. The median duration of the fenoldopam infusion was 115 h (IQR 76–302 h). The median starting dose was 0.3 mcg/kg/min (IQR 0.2–0.5 mcg/kg/min) and the median maximum dose was 0.6 mcg/kg/min (IQR 0.4–1.4 mcg/kg/min). In 8 cases, the infusion duration was less than 24 h.

### Fenoldopam efficacy

Fenoldopam was associated with a BP reduction (MAP, SBP and DBP) in all cases after 8 h of treatment, but in 87% of patients the MAP drop pressure after 8 h was higher than 25% of calculated drop pressure (Table 2). Normalization of MAP under the 95th percentile occurred in 26% and 35% of patients at 24 and 48 h, respectively. The median dose administered at these three time points was 0.3 mcg/kg/min (IQR 0.2–0.5 mg/kg/min), 0.4 mcg/kg/min (IQR 0.2–0.8 mg/kg/min) and 0.5 mcg/kg/min (IQR 0.3–1.0 mg/kg/min), respectively. MAP was normal in 77% of cases at the end of infusion.

MAP resulted in lower than the 95th percentile + 30 mmHg in 88% of cases at 8 h, 91% at 24 h, and 94% at 48 h. All hypertensive emergencies presented a resolution of symptoms within 8 h after the start of fenoldopam infusion. Neurological symptoms secondary to hypertension developed in only two cases during fenoldopam infusion (2%). No difference in terms of dose administered was observed in the first 8 h between hypertensive urgency and emergency (median fenoldopam dose of 0.3 (IQR 0.2–0.4) and 0.4 mcg/kg/min (IQR 0.2–0.5), respectively; *p* = 0.40).

In 14% of patients, fenoldopam was the only drug used to treat hypertension, 19% received one additional anti-hypertensive drug, and the remaining 67% were treated with two or more additional anti-hypertensive drugs. In 7 cases, a surgical treatment for hypertension resolution, such as kidney artery stenting or nephrectomy was required.

**Fig. 1:**
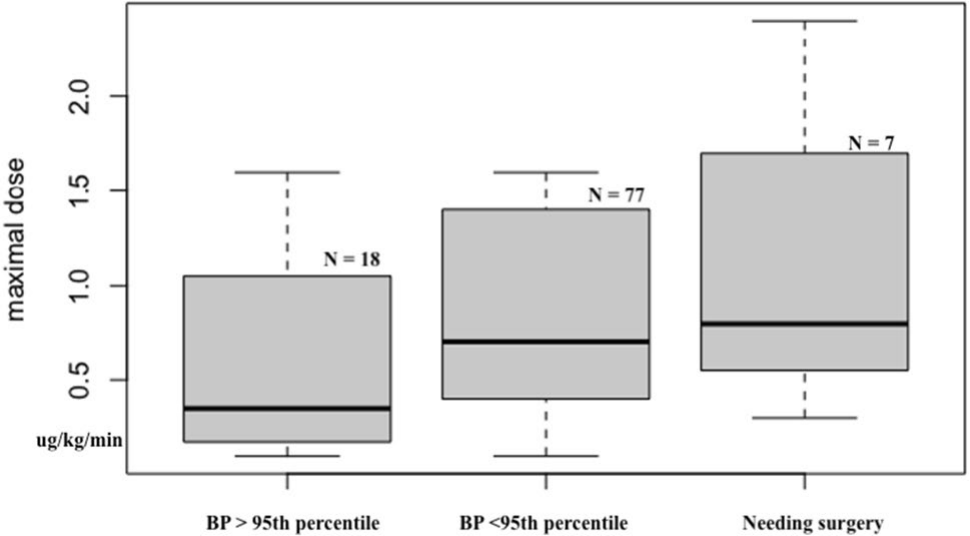
Differences in maximal dose of fenoldopam administered for patients with a mean arterial pressure > 95th percentile, < 95th percentile and who required a surgical correction of hypertension at the end of infusion

A maximal median dose of 0.8 mcg/kg/min (IQR 0.6–1.7 mcg/kg/min) of fenoldopam was administered in cases who required a surgical correction of hypertension, while the ones reaching a MAP less or higher than 95th percentile at the end of infusion received a maximal median dose of 0.7 (IQR 0.4–1.4 mcg/kg/min) and 0.35 mcg/kg/min (IQR 0.2–1.0 mcg/kg/min; *p* = 0.070), respectively (Fig. 1).

**Fig. 2:**
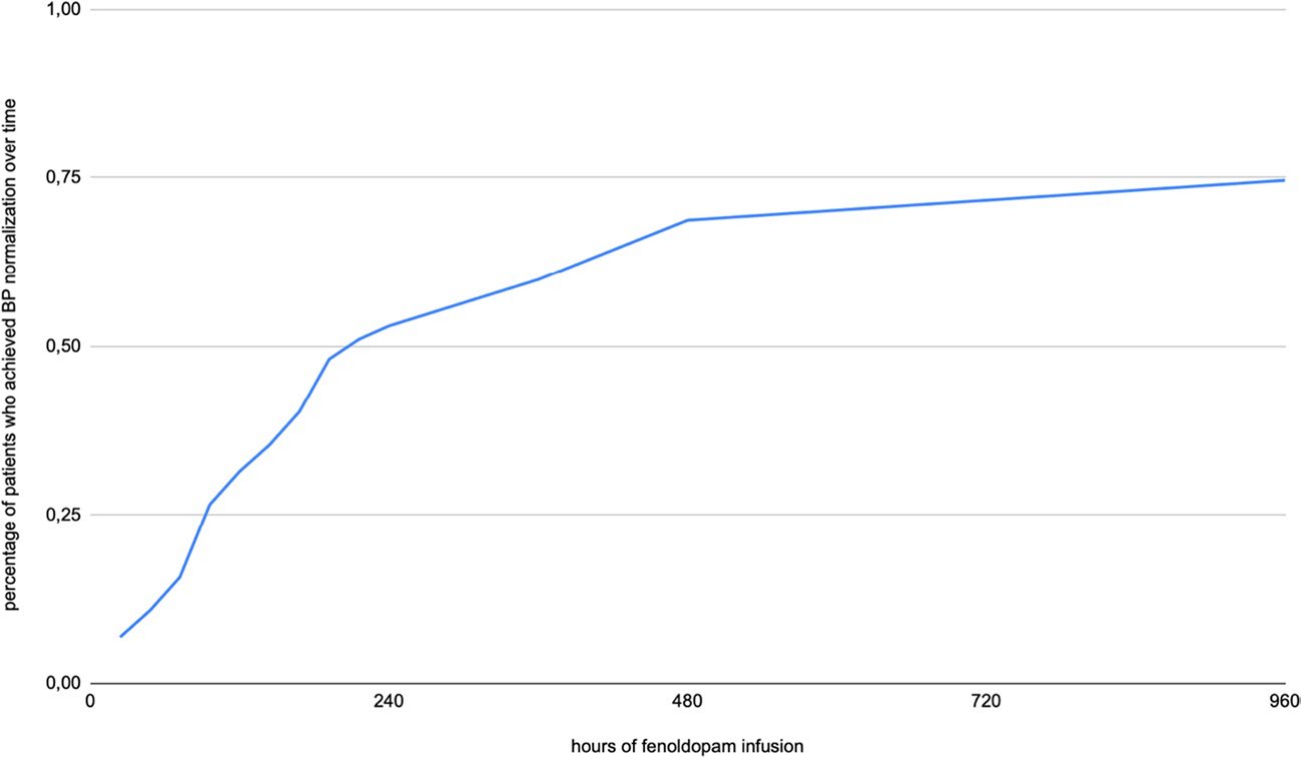
Percentage of patients who achieved BP normalization over time

The median duration of fenoldopam infusion to achieve BP normalization at the end of the infusion was 150 h (IQR 75–257 h). This duration did not significantly differ from that of patients who did not achieve BP normalization (median 144 h, IQR 90–300 h, *p* = 0.75), nor did it differ significantly from the time to surgical resolution of hypertensive crisis (median 346 h, IQR 137–515 h, *p* = 0.2). The percentage of patients who achieved BP normalization over time is presented in Fig. 2.

### Comparison between patients with or without BP drop > 25% at 8 h

**Fig. 3:**
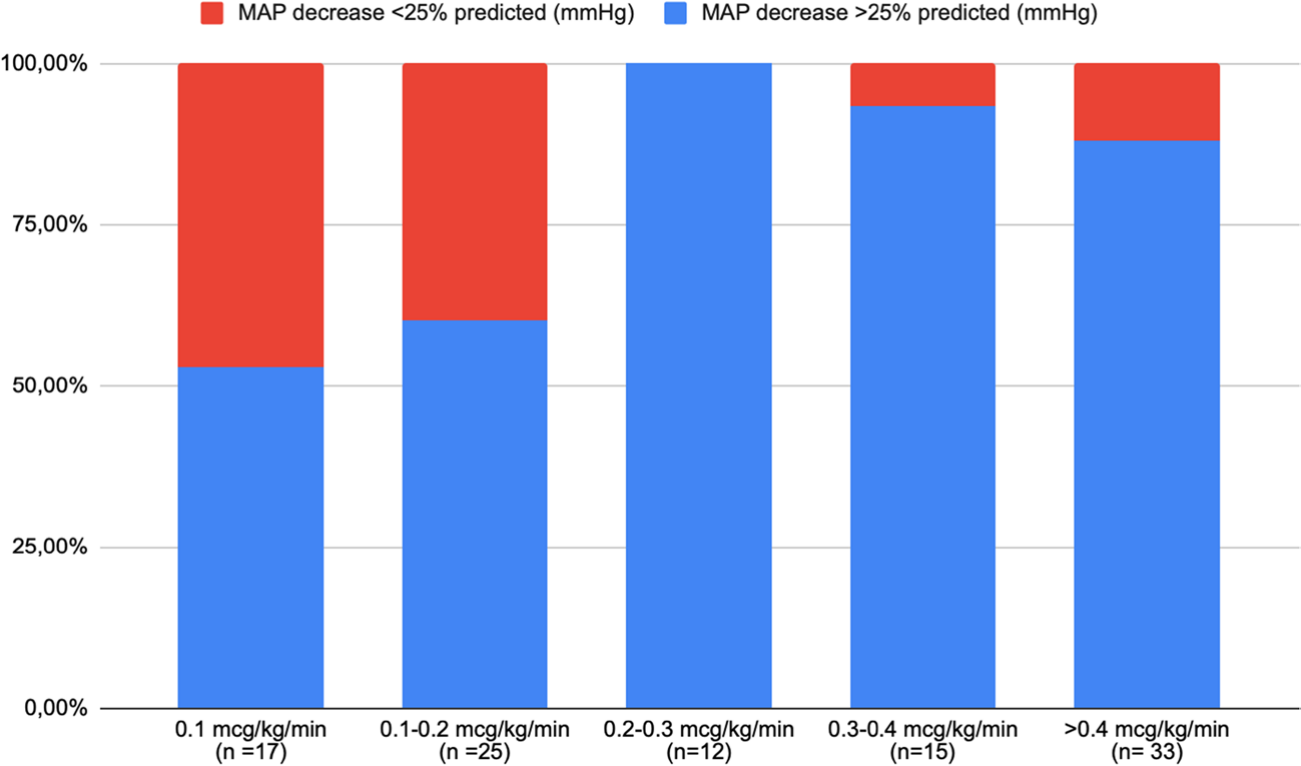
Distribution of cases that had a MAP reduction above or less than 25% of BP target according to fenoldopam dose after 8 h of treatment

**Fig. 4:**
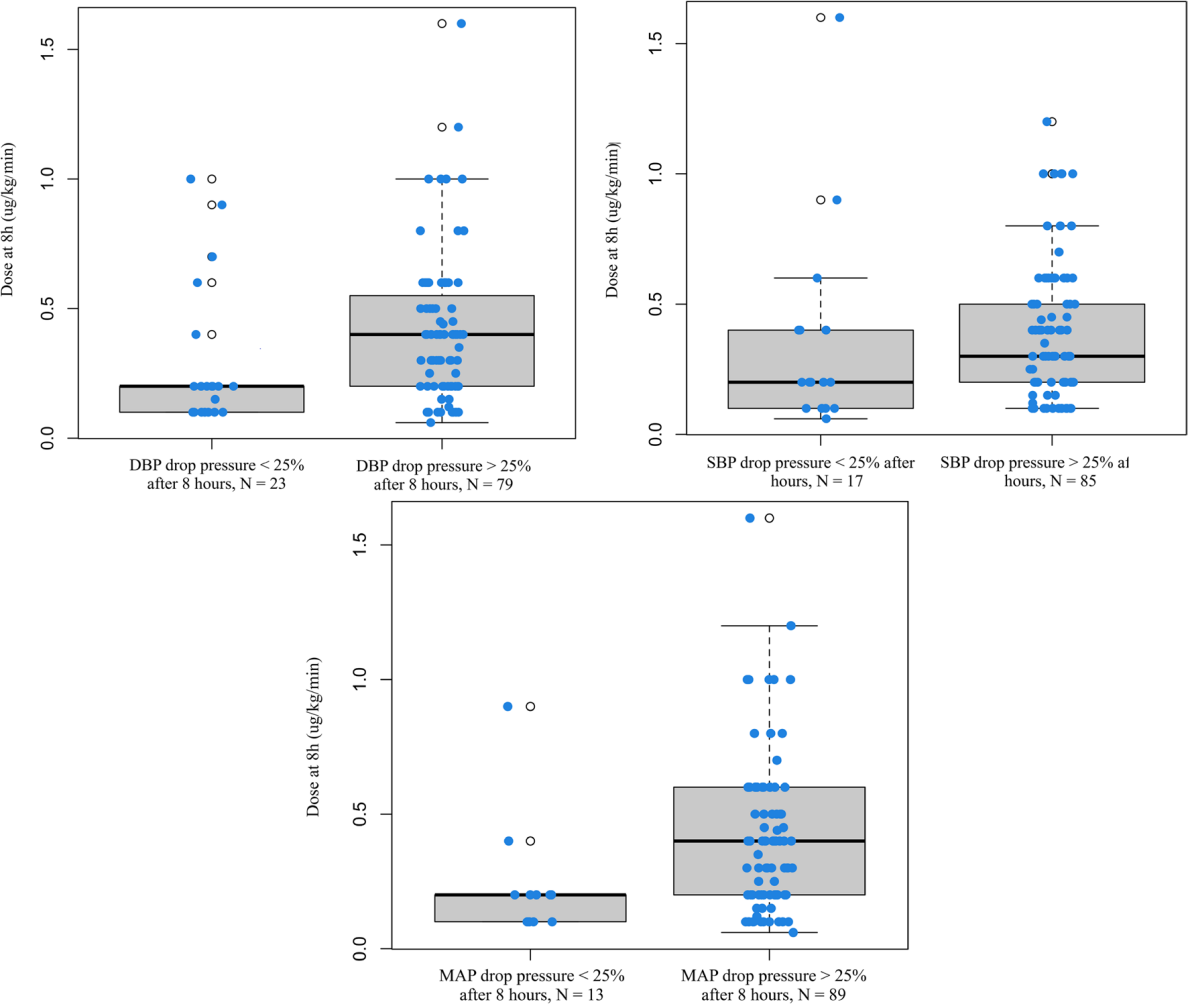
Differences in dose of fenoldopam administered for obtaining a DBP (left), SBP (right) and MAP (down) reduction exceeding or falling under the 25% of the target BP after 8 h. *SBP*: systolic blood pressure, *DBP*: dyastolic blood pressure, *MAP*: mean arterial pressure

Differences in fenoldopam dosages between patients having a BP reduction (SBP, DBP, MAP) exceeding or not the 25% of calculated drop after 8 h of infusion are presented in Table 3. Distribution of cases showing a MAP reduction exceeding or falling under the 25% of the target BP after 8 h of treatment, according to the administered fenoldopam dose, is presented in Fig. 3. Cases with a MAP reduction < 25% of calculated drop pressure received a lower median fenoldopam dose (0.2 mcg/kg/min; IQR 0.1–0.2) than patients having a MAP reduction > 25% of calculated drop pressure (0.4 mcg/kg/min; IQR 0.2–0.6; *p* = 0.002). The same results were obtained considering SBP and DBP (Fig. 4). A median fenoldopam dose not exceeding 0.2 mcg/kg/min at 8 h was associated with an acceptable reduction of MAP in 38% of cases. Conversely, higher dosages resulted in a MAP decrease exceeding 25% of the calculated target in 91% of patients (*p* < 0.001).

**Table 3:** Mean fenoldopam doses at 8, 24 and 48 h of infusion according to our study outcome measures

	Efficacy of fenoldopam at 8 h		
Fenoldopam infusions (*N* = 102)	MAP reduction > 25% target BP at 8 h	MAP reduction < 25% target BP at 8 h	p value
Mean dose of fenoldopam at 8 h (mcg/kg/min)	0.4 (IQR 0.2–0.6)	0.2 (IQR 0.1–0.2)	0.002*
	SBP reduction > 25% target BP at 8 h	SBP reduction < 25% target BP at 8 h	
Mean dose of fenoldopam at 8 h (mcg/kg/min)	0.325 (IQR 0.2–0.5)	0.2 (IQR 0.15–0.4)	0.023*
	DBP reduction > 25% target BP at 8 h	DBP reduction < 25% target BP at 8 h	
Mean dose of fenoldopam at 8 h (mcg/kg/min)	0.4 (IQR 0.2–0.55)	0.2 (IQR 0.1–0.2)	0.004*
Efficacy of fenoldopam at 24 h			
Fenoldopam infusions (*N* = 93)	MAP < 95°percentile at 24 h	MAP > 95°percentile at 24 h	*p*value
Mean dose of fenoldopam at 24 h (mcg/kg/min)	0.35 (IQR 0.2–0.5)	0.4 (IQR 0.2–0.8)	0.15
	SBP < 95°percentile at 24 h	SBP > 95°percentile at 24 h	
Mean dose of fenoldopam at 24 h (mcg/kg/min)	0.3 (IQR 0.2–0.5)	0.4 (IQR 0.2–0.9)	0.12
	DBP < 95°percentile at 24 h	DBP > 95°percentile at 24 h	
Mean dose of fenoldopam at 24 h (mcg/kg/min)	0.35 (IQR 0.2–0.55)	0.4 (IQR 0.2–0.8)	0.10
Efficacy of fenoldopam at 48 h			
Fenoldopam infusions (*N* = 89)	MAP < 95°percentile at 48 h	MAP > 95°percentile at 48 h	* p* value
Mean dose of fenoldopam at 48 h (mcg/kg/min)	0.5 (IQR 0.3–0.8)	0.5 (IQR 0.3–1.1)	0.40
	SBP < 95°percentile at 48 h	SBP > 95°percentile at 48 h	
Mean dose of fenoldopam at 48 h (mcg/kg/min)	0.5 (IQR 0.2–0.7)	0.5 (IQR 0.3–1.1)	0.42
	DBP < 95°percentile at 48 h	DBP > 95°percentile at 48 h	
Mean dose of fenoldopam at 48 h (mcg/kg/min)	0.4 (IQR 0.2–0.9)	0.5 (IQR 0.4–1.1)	0.48

*MAP* Mean arterial pressure, *SBP* Systolic blood pressure, *DBP* Diastolic blood pressure, *IQR* Interquartile range

### Comparison between patients with or without BP < 95th at 24 and 48 h

**Fig. 5:**
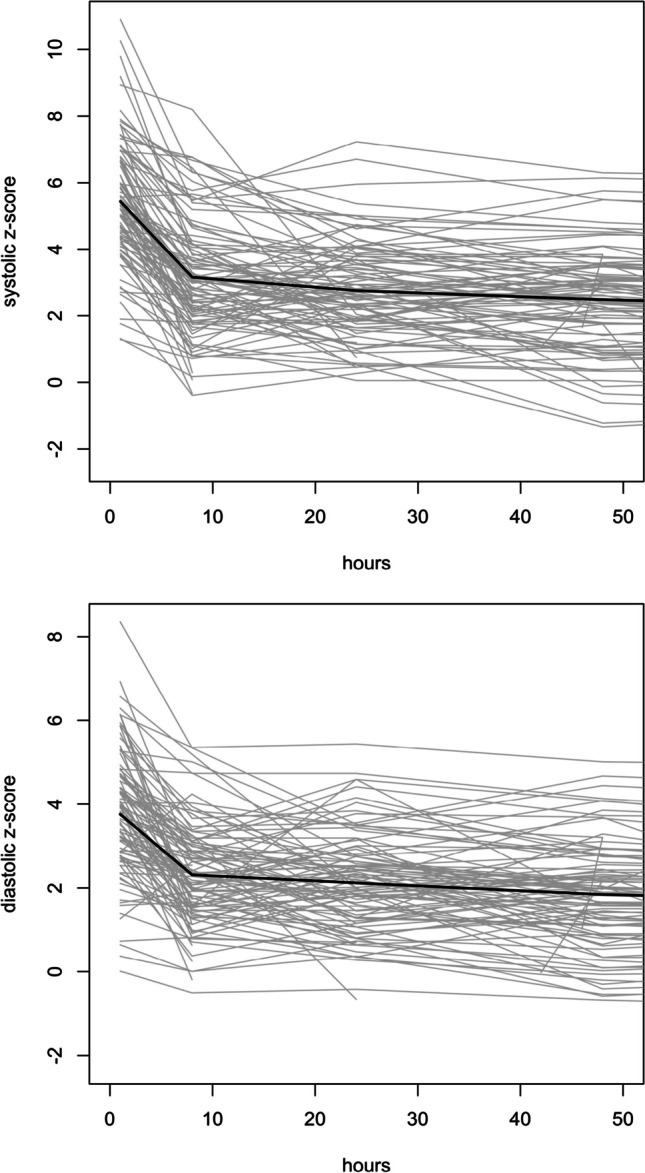
Standard deviation scores in systolic and diastolic blood pressure over time during the first 48 h of treatment with fenoldopam

Considering MAP normalization at 24 and 48 h of treatment, there were no differences in median fenoldopam doses between responders and non-responders (responders at 24 h 0.35 mcg/kg/min [IQR 0.2–0.5] vs. non-responders 0.4 mcg/kg/min [IQR 0.2–0.8], *p* = 0.150; responders at 48 h 0.5 mcg/kg/min [IQR 0.3–0.8] vs. non-responders 0.5 mcg/kg/min [IQR 0.3–1.1], *p* = 0.260). In multivariate analysis, no predictors of response to fenoldopam infusion were identified. The trend of both SBP and DBP SDS over time demonstrated a significant drop during the first 8 h of infusion, followed by a slower and progressive BP reduction until the end of infusion (Fig. 5).

### Fenoldopam safety

Hypotension developed in 6% of fenoldopam infusions after a median time of 164 h (IQR 36–418). Median fenoldopam doses were not significantly different in patients who experienced or not hypotension (0.8 mcg/kg/min IQR 0.5–0.95 in hypotension group vs. 0.7 mcg/kg/min IQR 0.4–1.4, *p* = 0.420). The administration of additional drugs also did not differ significantly between these two groups (*p* = 0.62), as reported in Table 4.

**Table 4:** Distribution of additional hypotensive drugs in cases that presented or not hypotension

Number of additional drugs	0	1	2 or more	Total
Hypotension group	1 (16.7%)	2 (33.3%)	3 (50.0%)	6 (100%)
No hypotension group	12 (12.5%)	19 (29.2%)	65 (67.7%)	96 (100%)

None of the patients with a MAP drop higher than 25% of calculated MAP reduction after 8 h of infusion presented symptoms of hypotension. Detailed drop of calculated MAP after 8 h was as follows: 46% of patients presented a reduction of 25–30%, 28% of cases of 30–35% and the remaining 13% presented a drop of 35–40%. Hypokalemia occurred in 13% of cases, 5% of patients presented nausea and 3% headache. No further adverse events were identified.

## Discussion

In this study, we explored the use of fenoldopam as a therapeutic approach for hypertensive crises in pediatric patients with AKI or CKD. Our study suggests that fenoldopam is effective in treating hypertensive urgencies and emergencies and an initial maximal dose of 0.2 mcg/kg/min should be administered in the first 8 h.

In terms of efficacy, both the American and European guidelines agree that, in treating a hypertensive crisis, BP reduction should not exceed 25% of the targeted decrease within the initial 8 h and that the complete normalization of BP should be achieved during 24–48 h [4, 8].

In our cohort, all patients presented a BP reduction after 8 h of treatment, but a BP drop exceeding 25% of target decrease was identified in 87% of cases, even if no patients required to stop the infusion due to hypotension signs. We demonstrated a dose-dependent efficacy in BP reduction during the first hours of treatment. A median fenoldopam dose not exceeding 0.2 mcg/kg/min at 8 h was associated with an adequate MAP reduction in almost 40% of cases, whereas higher dosages resulted in a MAP decrease exceeding 25% of the calculated target in more than 90% of patients. These findings prompted our center to adopt a more cautious approach to managing children with hypertensive crises, aiming at mitigating the excessive decrease in BP during the initial 8 h of treatment. However, it is also important to underline that 46% of patients presented a reduction of 25–30% of calculated MAP after 8 h and considering that none of our patients presented signs of hypotension in the first 8 h of infusion, probably a dose max of 0.2 mcg/kg/min in the first 8 h should be safe even if a strict BP monitoring is necessary.

Hammer et al. previously identified that a fenoldopam dose of 0.8 mcg/kg/min results in a substantial BP reduction in children undergoing anesthesia and surgery [21], even if according to our results the initial drop pressure with that dose is excessive. In our cohort, MAP normalization was observed in only 26% of cases after 24 h and in 35% after 48 h, but the median fenoldopam doses used in our patients were significantly lower than that proposed by Hammer and collegues. Moreover, 51% of our patients met the criteria for drug-resistant hypertension, as defined by the American guidelines. Indeed, BP SDS variation over time during infusion reveals a significant reduction in BP during the initial hours of treatment, followed by a reduced efficacy in achieving BP normalization. Such normalization might need a prolonged infusion, potentially spanning several days, and the introduction of additional medications. Conversely, the initial reduction in BP below the hypertensive urgency threshold affords a safe opportunity to manage these patients giving the time to identify the most suitable long-term therapeutic regimen.

Despite its potential as an antihypertensive agent in adults, the response to fenoldopam in children appears variable [21, 24, 25]. Dopamine receptor system in children may be less developed compared to adults. Moreover, children might have a different drug metabolism, potentially necessitating higher doses than those established for adults [26]. Two of the largest studies investigating the use of fenoldopam for BP control in children to limit blood loss during surgical procedures, reported an effective drug dosage range notably higher in pediatric patients undergoing anesthesia and surgery (0.8–1.2 μg/kg/min) compared to the range reported for adults (0.05–0.3 μg/kg/min) [17, 26]. However, the recommended maximum dosage is 0.8 mcg/kg/min in children, in contrast to a maximum of 1.6 mcg/kg/min in adults [27].

These discrepancies highlight the importance of tailored approaches to pediatric hypertension management and emphasizes the necessity of more targeted research to elucidate the effectiveness of fenoldopam in children.

In studies focusing on pharmacodynamic and pharmacokinetic properties involving a fixed fenoldopam dose, the incidence of adverse events was increased when dosages were higher than 0.8 mcg/kg/min [18, 27–29]. In our study population, we observed mild side effects with hypotension occurring in 6% of patients, which resolved in all cases upon discontinuation of the infusion. Of note, no patients with a median BP drop > 25% demonstrated any clinically evident side effects. Notably, the median dose of fenoldopam in these cases was 0.8 mcg/kg/min, which aligns with previous research, although it was comparable to patients who did not experience hypotension. Hypokalemia was observed in 13% of cases, a result that is in line with previous findings in studies in adults [28]. However, in this specific population of patients, several factors might contribute to inducing hypokalemia, including the use of loop diuretics or potentially inadequate potassium replacement in intravenous fluids.

Our study has several limitations. It is a retrospective analysis, which means that it relies on a review of medical charts to retrieve data that may be subjected to reporting bias. Additionally, this study is observational, offering insights into real-world scenarios but without the controlled conditions of a clinical trial. Furthermore, the study involves a single cohort of patients, which may limit the generalizability of the findings to a broader population. The overall sample size is relatively small, and it is important to note that 67% of patients received two or more anti-hypertensive medications during fenoldopam infusion, introducing potential confounding variables that could affect results. These limitations underline the need for further research and the cautious interpretation of the study's outcomes. Comparative studies, specifically those comparing fenoldopam with other antihypertensive agents, would be invaluable in enhancing our understanding of the most effective and safe treatments for hypertensive crises in this pediatric population.

## Conclusions

This study provides compelling evidence to suggest that fenoldopam is an effective and well-tolerated medication for the initial management of hypertensive crises in children afflicted by kidney conditions. An initial maximal dose of 0.2 mcg/kg/min should be administered in the first 8 h maintaining a strict BP monitoring in order to identify a possible excessive drop pressure in the first hours of infusion. Further prospective and multicenter investigations are necessary to confirm our preliminary results.

## Supplementary Information

Below is the link to the electronic supplementary material.

Graphical abstract (PPTX 225 kb)

## Data Availability

The data that support the findings of this study are available on request from the corresponding author (NBP). The data are not publicly available due to restrictions.

## Declarations

### Board approval

The present study is approved by the Ethics Committee of Padua with the number of protocol 348n/AO/23.
