# Supplementary material for: Efficacy and safety of fenoldopam for the treatment of hypertensive crises in children with kidney disease: a retrospective study
Source: Pediatr Nephrol. 2024 Sep 9;40(1):165–75. doi: 10.1007/s00467-024-06490-7 (PMC11584497; doi:10.1007/s00467-024-06490-7)
Supplement: Supplementary file 1 — Graphical abstract (PPTX 225 kb) [file 467_2024_6490_MOESM1_ESM.pptx]

## Slide 1
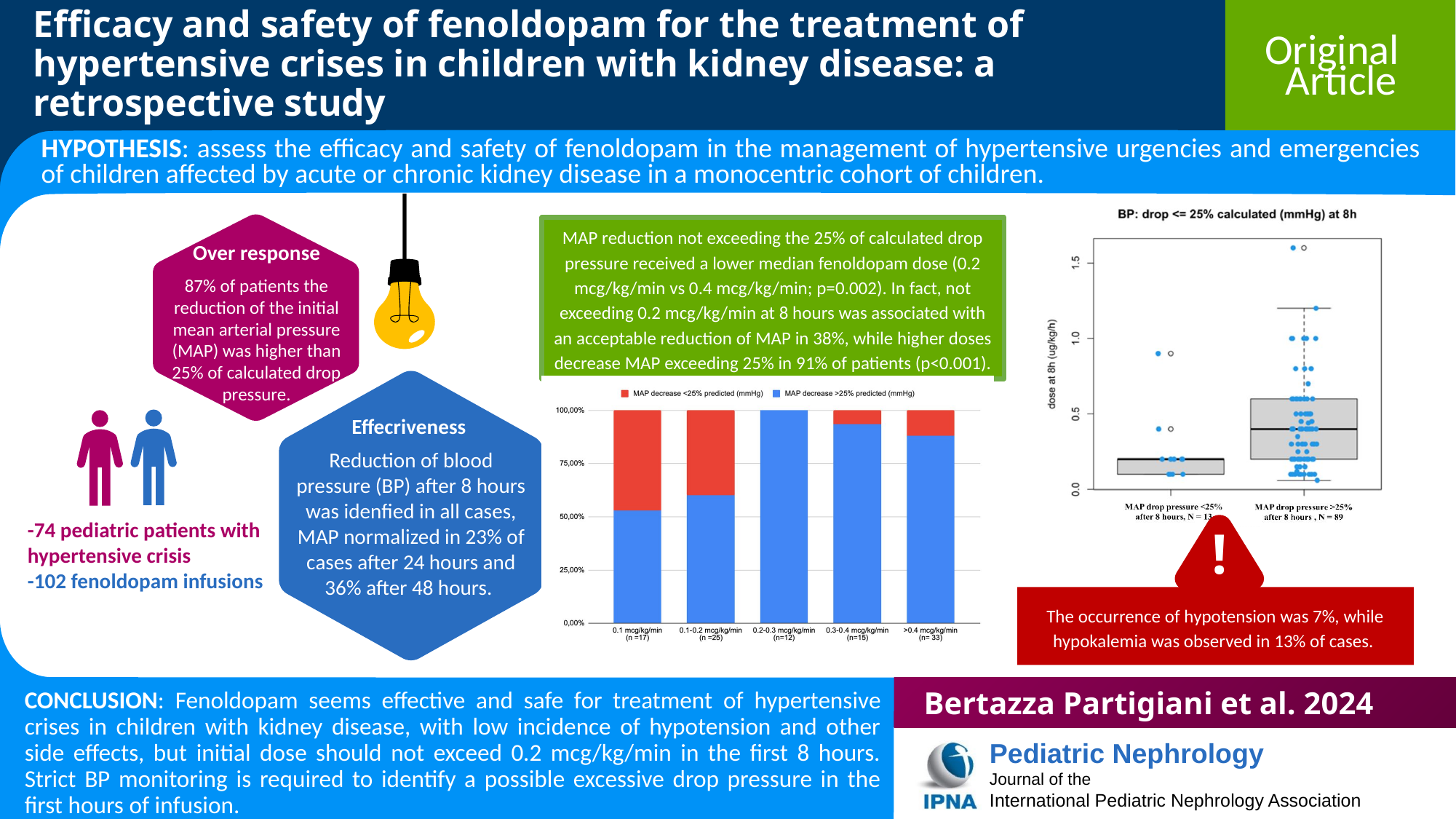

Efficacy and safety of fenoldopam for the treatment of hypertensive crises in children with kidney disease: a retrospective study
HYPOTHESIS: assess the efficacy and safety of fenoldopam in the management of hypertensive urgencies and emergencies of children affected by acute or chronic kidney disease in a monocentric cohort of children.
MAP reduction not exceeding the 25% of calculated drop pressure received a lower median fenoldopam dose (0.2 mcg/kg/min vs 0.4 mcg/kg/min; p=0.002). In fact, not exceeding 0.2 mcg/kg/min at 8 hours was associated with an acceptable reduction of MAP in 38%, while higher doses decrease MAP exceeding 25% in 91% of patients (p<0.001).
Over response
87% of patients the reduction of the initial mean arterial pressure (MAP) was higher than 25% of calculated drop pressure.
Effecriveness
Reduction of blood pressure (BP) after 8 hours was idenfied in all cases, MAP normalized in 23% of cases after 24 hours and 36% after 48 hours.
-74 pediatric patients with hypertensive crisis
-102 fenoldopam infusions
!
The occurrence of hypotension was 7%, while hypokalemia was observed in 13% of cases.
Bertazza Partigiani et al. 2024
CONCLUSION: Fenoldopam seems effective and safe for treatment of hypertensive crises in children with kidney disease, with low incidence of hypotension and other side effects, but initial dose should not exceed 0.2 mcg/kg/min in the first 8 hours. Strict BP monitoring is required to identify a possible excessive drop pressure in the first hours of infusion.
